# Subacromial injection of hydrolyzed collagen in the symptomatic treatment of rotator cuff tendinopathy: an observational multicentric prospective study on 71 patients

**DOI:** 10.1016/j.jseint.2023.06.009

**Published:** 2023-07-07

**Authors:** Matteo Buda, Sabri Dlimi, Marco Parisi, Andrea Benoni, Gianluca Bisinella, Stefano Di Fabio

**Affiliations:** a Department of Orthopaedics and Trauma Surgery, Ospedali Riuniti Padova Sud, Monselice, (PD), Italy; c Department of Orthopaedics and Trauma Surgery, Poliambulatorio Arcella, Padova, Italy; d Department of Orthopaedics and Trauma Surgery, San Martino Hospital of Belluno, ULSS1 Dolomiti, Belluno, Italy; e Department of Orthopaedics and Trauma Surgery, University of Verona, Verona, Italy; b Department of Orthopedics and Trauma Surgery Rizzoli-Argenta, Istituto Ortopedico Rizzoli, Argenta, (FE), Italy

**Keywords:** Tendinopathy, Rotator cuff, Infiltrative device, Hydrolyzed collagen, Tendons, Non-pharmacological therapy

## Abstract

**Background:**

The purpose of the present observational multicentric prospective study was to evaluate the efficacy and safety of a new infiltration device (CHondroGrid, Bioteck S.p.A, Arcugnano, Italy) based on hydrolyzed collagen in the treatment of rotator cuff tendinopathy.

**Methods:**

Seventy-one patients (53.3 ± 11.6 years old) affected by rotator cuff tendinopathy were treated in 2021 with two subacromial injections of CHondroGrid at 13 ± 2.9 days apart. The outcomes measured were the visual analog scale (VAS) score (in movement, during the night, and at rest), the Constant Score, the Simple Shoulder Test, and patient satisfaction. The outcome variables were measured before each injection, at 1 month and at 6 months after the last injection.

**Results:**

The treatment was significantly effective from the first injection and up to the six-month follow-up. At the last follow-up, the VAS score on movement had improved by 71% (*P* < .001), while the VAS score at rest and during the night had ameliorated by 91% and 87%, respectively (*P* < .001). The Constant Score and Simple Shoulder Test improved by 32% and 61%, respectively (*P* < .001). No adverse events were reported.

**Conclusions:**

CHondroGrid resulted in a safe and effective treatment in pain relief and for the functional recovery of rotator cuff tendinopathy.

Rotator cuff tendinopathies (RCTs) are among the most frequent pathologies causing pain and functional deficits in the shoulder.^17^^,^^19^ The most recent studies have shown that RCT can be multifactorial, since both intrinsic and extrinsic factors (ie, the disorganization of the extracellular matrix (ECM) collagen, apoptosis, necrosis, and changes in cell morphology) play an important role, with a significant incidence over age 50.^13^^,^^18^^,^^20^, ^21^, ^22^, ^23^

To date, physiotherapy, nonsteroidal anti-inflammatory drugs, periarticular injections of hyaluronic acid (HA) and/or corticosteroids, platelet-rich plasma injections, prolotherapy, and local microwave diathermy are considered among the most effective conservative treatments in the management of RCT.^17^ However, the effectiveness of these therapies is still controversial.^11^ Therefore, there is a need to identify new therapeutic options with different and effective mechanisms of action.

In recent years, the use of collagen as an alternative approach to more traditional products for the treatment of painful symptoms and loss of functionality of the joints and muscle-tendon and ligament structures has been attracting the interest of researchers.^7^^,^^8^^,^^12^

Recently, a novel injectable collagen formulation consisting in type I hydrolyzed bovine collagen with a low molecular weight (<3 kDa) (CHondroGrid, Bioteck S.p.A, Arcugnano, Italy) was introduced for the treatment of joint pain and functional recovery through intra-articular injections, with excellent results being reported.^4^^,^^24^

In the literature, no studies investigating the clinical outcomes in the treatment of RCT with hydrolyzed collagen injections have been published so far.

The aim of the present study is to investigate the efficacy and safety of subacromial injections of CHondroGrid in reducing pain and promoting functional recovery in patients with RCT.

## Materials and methods

This multicentric, observational prospective study was approved by the Ethical Committee for clinical investigation of Padua (Study ID:20758) and an informed consent was obtained from each patient. The study was conducted in agreement with the Helsinki Declaration. Two centers participated to the study: Poliambulatorio Arcella (Padua, Italy) and Orthopedics and Traumatology Department, San Martino Hospital (Belluno, Italy). All consecutive patients who referred to each of the participating centers between January 2021 and December 2021, and who were treated with periarticular, subacromial injections of CHondroGrid for painful shoulder, were screened for eligibility.

Inclusion criteria were persistent shoulder pain for at least three months, unresponsive to conservative treatments (nonsteroidal anti-inflammatory drugs and/or physiotherapy), and clinical diagnosis of RCT or partial thickness tear detected with high field magnetic resonance imaging.

Exclusion criteria were previous treatment with subacromial injections of steroids or HA in the previous three months, history of shoulder trauma, age < 18 years old and > 70 years old, complete RC tears, calcifying tendinitis, previous arthroscopic or open shoulder surgery, shoulder instability, adhesive capsulitis, infections or neoplasm, symptomatic cervical spine disease, rheumatoid arthritis or immune diseases, gout and uric acid diseases, severe medical conditions, pregnancy, denied informed consent.

CHondroGrid was prepared accordingly to its instruction for use, namely dissolving the 4 mg of product provided in 2 ml of water for injection. All patients received two 2 ml (4mg) ultrasound-guided CHondroGrid injections in the subacromial bursa just above the involved tendon through a lateral or posterior approach. The second injection was performed between 9 and 17 days after the first one (mean: 13 days, standard deviation: 2.9 days).

The main measured outcomes were changes in pain severity and functional recovery. Secondary outcomes were patient satisfaction and adverse events.

The following variables were collected: demographic data (age, gender, body mass index, dominant arm) and medical history (chronic diseases and onset of symptoms); pain severity and functional disability according to the visual analog scale (VAS) score measured at rest, moving, and during the night, Constant Score (CS), and Simple Shoulder Test (SST) recorded just before the first (baseline/T0) and the second (T1) CHondroGrid injections as well as at one-month (T2) and six-month (T3) follow-ups from the last injection; the percentage of patient satisfaction was reported at a six-month follow-up after the last injection.

All patients provided their informed consent to treatment with CHondroGrid. The authors followed the ‘Strengthening the Reporting of Observational Studies in Epidemiology (STROBE)’ statement guidelines for observational cohort studies.^5^

### Statistical analysis

Statistical analysis was conducted using R (R Core Team version 4.0.5; R Foundation for Statistical Computing, Vienna, Austria). The Kolmogorov-Smirnov normality test was used to examine if variables were normally distributed. Normally distributed continuous data were reported as mean ± standard deviation and categorical variables as numbers and percentages. Comparisons of normally distributed variables were performed using unpaired *t-*tests. Multiple data comparisons on VAS, CS, and SST at different time points (T0-T3) were performed with the analysis of variance for repeated measures, and the Bonferroni post hoc test was used when indicated. All statistical tests were 2-tailed and statistical significance was defined as *P* < .001.

## Results

A total of 73 patients referred to one of the participating centers were treated with periarticular injections of CHondroGrid for painful shoulder during the study period. Among them, two patients were lost to follow-up: one decided to undergo surgery and another received a corticosteroid injection and was, as such, excluded. Therefore, 71 patients were finally included in the analysis.

The mean age of the study population was 53.3 ± 11.6 years and 43 (61.6%) patients were female. In 61% (42 patients), the dominant arm was affected by tendinopathy and treated with CHondroGrid. Baseline demographic and clinical characteristics are presented in Table I.

**Table I:** Baseline demographic and clinical characteristics of the study population.

Population parameters	Total patients n = 71
Demographic	
Age (y), mean (SD)	53.30 (11.59)
Gender (male), n (%)	28 (39.4)
Body mass index (kg/m^2^), mean (SD)	24.91 (3.97)
Dominant arm, n (%)	42 (61)
Right shoulder, n (%)	48 (67.6)
Smoking, n (%)	14 (20)
Comorbidities	
Hypertension, n (%)	29 (40.8)
Diabetes, n (%)	5 (7.0)
Hypothyroidism, n (%)	11 (15.5)
Metabolic syndrome, n (%)	17 (26.2)

*SD,* standard deviation.

**Figure 1:**
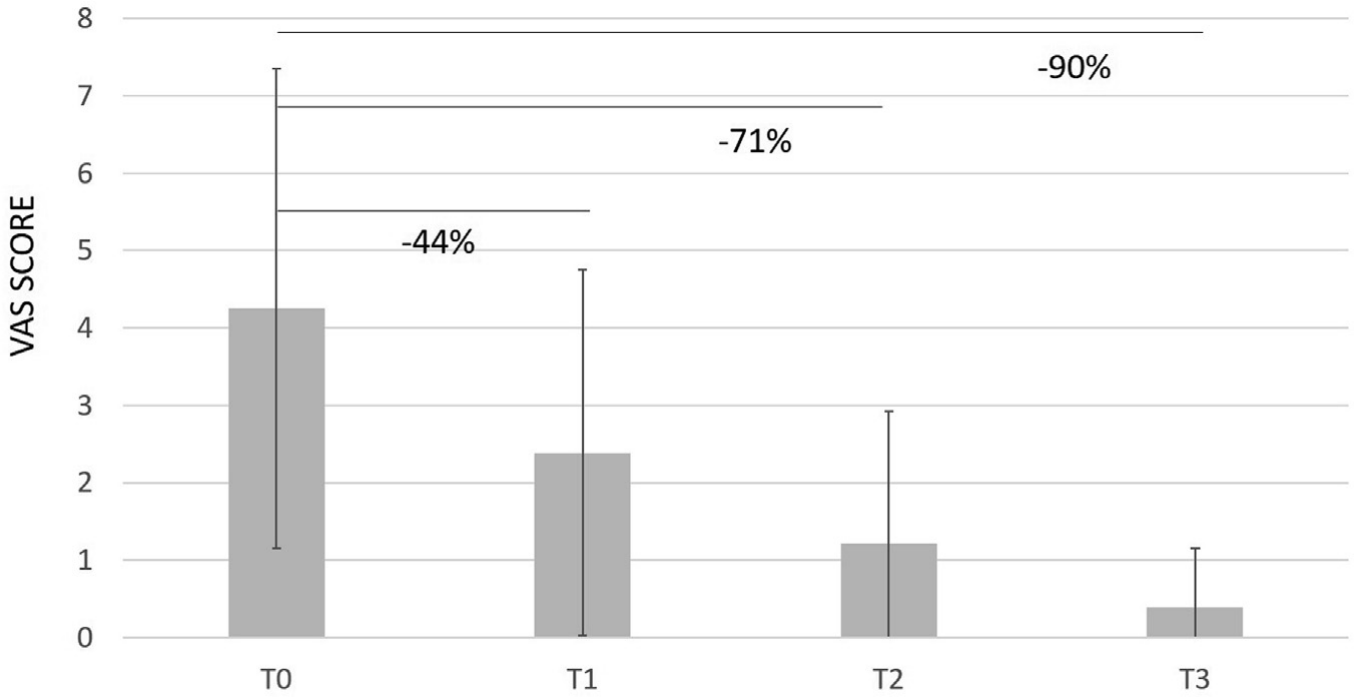
VAS at rest. The percentages refer to the reduction of pain relative to T0. *VAS,* visual analog scale.

**Figure 2:**
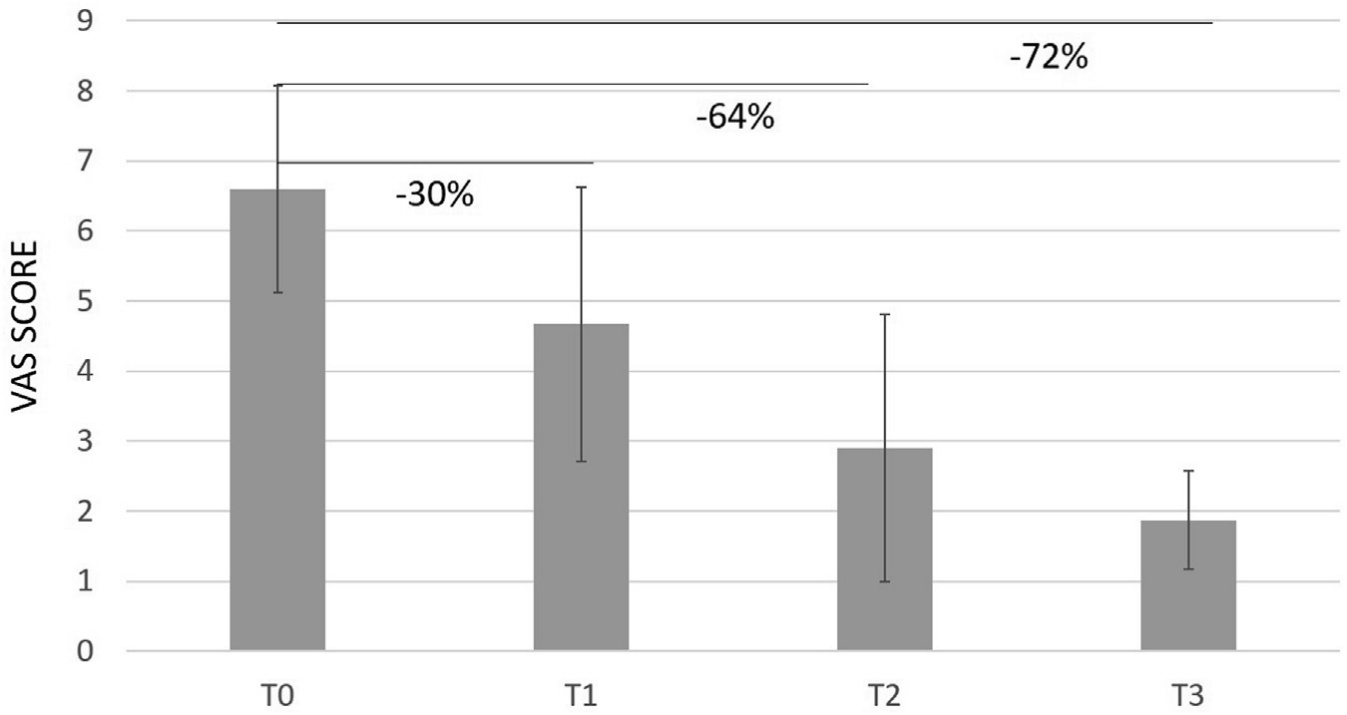
VAS during movement. The percentages refer to the reduction of pain relative to T0. *VAS,* visual analog scale.

**Figure 3:**
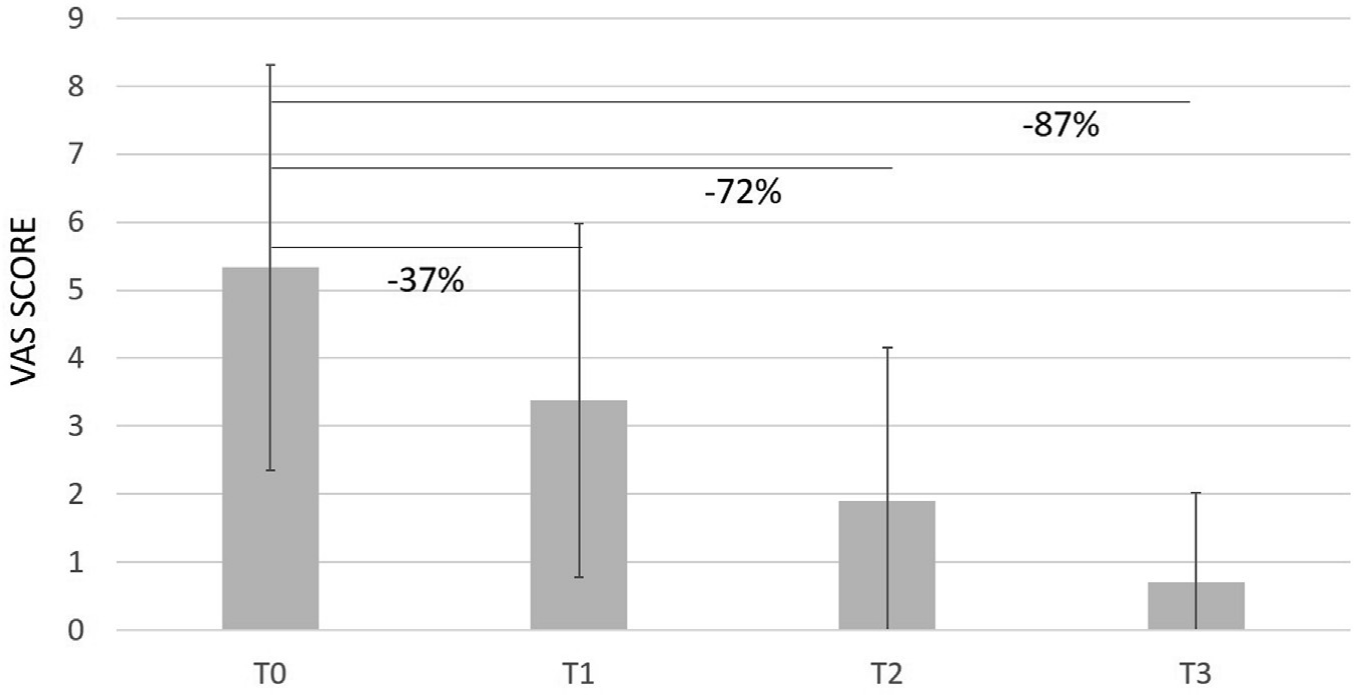
VAS during night. The percentages refer to the reduction of pain relative to T0. *VAS,* visual analog scale.

Overall, CHondroGrid significantly reduced pain severity and improved functional recovery from the first injection. In detail, pain severity at rest was 44% lower after the first application and progressively improved, reducing by up to 71% and 91% at the 1-month and six-month follow-ups, respectively (Table II; Fig. 1). Similarly, the VAS during movement and at night lowered by 30% and 37%, respectively, after the first injection. At the 1-month follow-up, the VAS during movement and at night was, respectively, 64% and 72% lower. At the 6-month follow-up, the VAS during movement and at night had reduced by 71% and 87%, respectively (Table II; Figure 2, Figure 3 and Figure 2, Figure 3).

**Table II:** Differences in VAS, Constant, and Simple Shoulder Test scores at different time points.

Outcomes measured	T0	T1	Difference between T0 and T1	T2	Difference between T1 and T2	T3	Difference between T2 and T3	% Improvement T0-T3
**Overall population**								
VAS at rest, mean (SD)	4.25 (3.10)	2.39 (2.37)	***P* < .001**	1.22 (1.71)	***P* < .001**	0.39 (0.77)	***P* < .001**	**91%**
VAS during movement, mean (SD)	6.56 (1.47)	4.59 (1.88)	***P* < .001**	2.39 (2.37)	***P* < .001**	1.87 (1.85)	***P* < .001**	**71.5%**
VAS during night, mean (SD)	5.33 (2.98)	3.38 (2.60)	***P* < .001**	1.90 (2.25)	***P* < .001**	0.70 (1.32)	***P* < .001**	**87%**
Constant Score, mean (SD)	63.76 (12.50)	71.45 (13.66)	***P* < .001**	80.81 (11.69)	***P* < .001**	84.07 (11.47)	***P* < .001**	**32%**
Simple Shoulder Test, mean (SD)	54.14 (20.16)	64.19 (23.83)	***P* < .001**	79.75 (18.50)	***P* < .001**	87.15 (14.99)	***P* < .001**	**61%**
**Group 1 (23 patients) with Simple Shoulder Test ≤ 42**								
VAS at rest, mean (SD)	6.35 (2.29)	4.09 (2.31)	***P* < .001**	2.09 (1.56)	***P* < .001**	0.86 (0.99)	***P* < .001**	**87%**
VAS during movement, mean (SD)	7.26 (1.32)	5.65 (1.46)	***P* < .001**	3.43 (2.04)	***P* < .001**	1.77 (1.87)	***P* < .001**	**76%**
VAS during night, mean (SD)	6.56 (2.48)	4.48 (2.66)	***P* < .001**	2.74 (2.14)	***P* < .001**	0.91 (1.27)	***P* < .001**	**86%**
Constant Score, mean (SD)	51.52 (10.90)	59.17 (13.63)	***P* < .001**	71.83 (9.37)	***P* < .001**	75.10 (10.06)	***P* < .001**	**46%**
Simple Shoulder Test, mean (SD)	30.43 (12.20)	40.58 (22.79)	***P* < .001**	63.40 (15.84)	***P* < .001**	77.27 (16.70)	***P* < .001**	**154%**
**Group 2 (28 patients) with Simple Shoulder Test ≥ 43 and ≤ 74**								
VAS at rest, mean (SD)	4.28 (3.01)	2.07 (2.16)	***P* < .001**	1.14 (1.94)	***P* = .009**	0.18 (0.56)	***P* = .02**	**96%**
VAS during movement, mean (SD)	6.57 (1.26)	3.96 (2.05)	***P* < .001**	2.93 (2.40)	***P* = .002**	1.89 (1.82)	***P* = .01**	**71%**
VAS during night, mean (SD)	5.03 (2.77)	3.04 (2.28)	***P* < .001**	2.11 (2.53)	***P* = .06**	0.59 (1.15)	***P* = .002**	**88%**
Constant Score, mean (SD)	65.32 (6.57)	74.10 (8.45)	***P* < .001**	81.36 (10.55)	***P* < .001**	85.37 (10.24)	***P* = .03**	**31%**
Simple Shoulder Test, mean (SD)	56.79 (6.75)	71.58 (16.06)	***P* < .001**	83.33 (15.88)	***P* < .001**	90.74 (13.34)	***P* = .003**	**60%**
**Group 3 (20 patients) with Simple Shoulder Test ≥ 75**								
VAS at rest, mean (SD)	1.90 (2.36)	0.95 (1.47)	***P* = .02**	0.35 (0.99)	***P* = .05**	0.15 (0.49)	***P* = .41**	**92%**
VAS during movement, mean (SD)	5.85 (1.53)	4.30 (1.62)	***P* < .001**	2.25 (1.62)	***P* < .001**	1.90 (1.97)	***P* = .43**	**67.5%**
VAS during night, mean (SD)	4.55 (3.41)	2.75 (2.63)	***P* < .001**	0.75 (1.41)	***P* < .001**	0.65 (1.63)	***P* = .74**	**86%**
Constant Score, mean (SD)	75.10 (7.64)	81.85 (8.21)	***P* = .002**	90.55 (7.18)	***P* < .001**	92.45 (7.19)	***P* = .22**	**23%**
Simple Shoulder Test, mean (SD)	77.49 (3.91)	81.24 (8.92)	***P* = .07**	94.18 (7.68)	***P* < .001**	94.18 (7.69)	***P* = .99**	**21.5%**

*SD,* standard deviation; *VAS,* visual analog scale; *T0,* at baseline; *T1,* before the second injection; *T2,* at 1 month from the second injection; *T3,* at six months from the second injection.

**Figure 4:**
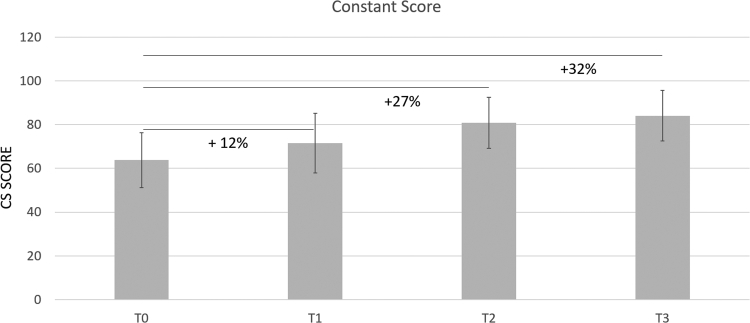
Constant Score. The percentages refer to the improvement of shoulder functionality relative to T0.

**Figure 5:**
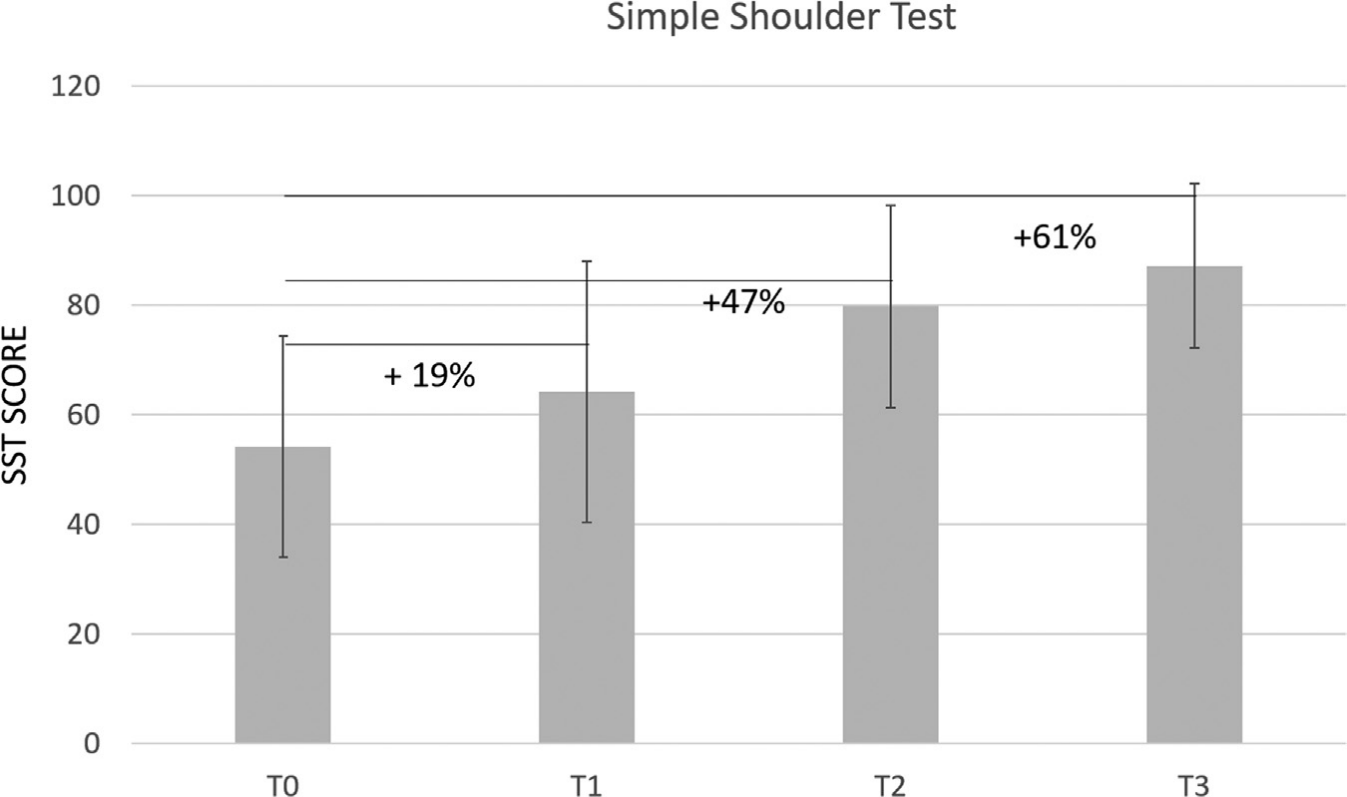
Simple Shoulder Test. The percentages refer to the improvement of shoulder functionality relative to T0.

Concurrently, CS and SST improved by 12% and 19% after the first treatment, and up to 21% and 32% at the 1-month follow-up. At the six-month follow-up, the CS and SST had improved by 32% and 61%, respectively (Table II; Figure 4, Figure 5 and Figure 4, Figure 5). A noteworthy consideration is that the improvements of all scores are significant between each time point. The average patient satisfaction at the six-month follow-up was 84 %.

The whole population comprised patients with different grades of initial pain that had a different initial functionality, which was measured by the SST. We divided the whole population into three groups in order to analyze if CHondroGrid had the same efficacy in the improvement of functionality and pain relief in all types of patients. In particular, we defined a group 1 (23 patients, SST score <42, low initial functionality), a group 2 (28 patients, 43<SST<74, medium initial functionality), and a group 3 (20 patients, SST>74, high initial functionality). In each group, we analyzed the % of improvement at each time point of the measured variables (SST, CS, and VAS scores) (see Table II). Interestingly, we observed that at the six-month follow-up, in group 1, the SST score ameliorated by 154%, while in groups 2 and 3, the SST score improved by 60% and 22%, respectively. Regarding the CS, a trend similar to that of the SST score was observed: in group 1, the CS Score ameliorated by 46 %, while in groups 2 and 3 the CS Score improved by 31% and 23%, respectively. The improvement of VAS pain at rest, in movement, and during the night was similar in all groups. No serious adverse events were reported. A mild local skin reaction due to the infiltrative procedure and not related to the device itself was observed in two patients. Such reaction lasts about 1 hour and was treated by using an ice bag on the site of injection for 20 minutes.

## Discussion

The present clinical study is the first study ever published on the efficacy and safety of the use of hydrolyzed collagen in RCT. RCT is the most common tendon disorder of the shoulder, causing pain and dysfunction.^23^ RCT has a multifactorial etiology (ie, genetic influence, hypoxia, exogenous substance, and hormones) that results in the disorganization and detrimental changes of the tendon ECM by increasing collagen III expression and cellular apoptosis events.^18^ In fact, ECM is a complex tissue composed mainly of water, proteoglycans, and collagen.^3^ In particular, type I collagen is the main component of the rotator cuff tendons and constitutes 85% of its dry weight.^3^^,^^23^ In the past decade, commonly used treatments for lowering RCT pain involved facilitating tendon sliding (HA), or using pharmaceuticals with an antinociceptive effect (corticosteroids), or stimulating metabolic activity through electromagnetic waves (diathermy).^17^

The results obtained with hydrolyzed collagen are encouraging. Indeed, our data indicate a pain reduction and functional recovery already after the first injection. The treatment effect increased progressively with the therapy and appeared to be still ongoing even after 6 months from the last injection. At the six-month follow-up, there was a pain reduction of > 70% during movement and of 91% and 87%, respectively, at rest and during the night. In addition, the CS and SST had progressively improved by up to 32% and 61% after six months. The results here presented show that CHondroGrid is effective also in patients with low initial functionality. Indeed, by dividing the whole study population into 3 groups (low initial functionality, medium initial functionality, and high initial functionality), the SST score at six months had improved by 154% in patients with low initial functionality (Table II). In the other two groups, at the six-month follow-up, the SST score had improved by 58% (patients with medium initial functionality) and 22% (patients with high initial functionality), respectively. Furthermore, although the three groups had a slightly different initial VAS score, they displayed a similar % of improvement (see Table II). These results show that the treatment with hydrolyzed collagen was able to both reduce pain and support a functional recovery of patients with different grades of initial shoulder functionality impaired by RCT. No serious adverse events were reported. A mild local skin reaction due to the infiltrative procedure and not related to the device itself was observed in two patients. Such reaction lasts about 1 hour and was treated by using an ice bag on the site of injection for 20 minutes. Moreover, patients claimed to be satisfied with treatment.

The results on periarticular use of CHondroGrid presented here are in agreement with the data available on the intra-articular use of CHondroGrid. Indeed, two observational studies have demonstrated its safety and efficacy in the treatment of 90 patients with knee osteoarthritis (OA).^4^^,^^24^ In these studies, the intra-articular use of CHondroGrid improved the pain, measured with VAS and Lequesne scores, by over 50%, and according to the Western Ontario and McMaster Universities Osteoarthritis Index by 80%, even more than 6 months after the last injection.^4^^,^^24^

Considering that pain reduction is achieved without significant adverse effects, the treatment with hydrolyzed collagen is a valid alternative to corticosteroids. Indeed, although intra-articular corticosteroid injections greatly reduce the risk of systemic undesirable reactions while still obtaining local anti-inflammatory effects, the numerous and potentially severe complications of corticosteroid-induced adverse events should not be underestimated.^6^ In addition, it has been reported that injections of corticosteroids may lead to cartilage and tendon damage.^2^

Other types of treatment based on collagen, following the promising results of *in vitro* and preclinical studies,^14^, ^15^, ^16^ have been successfully introduced in the treatment of knee OA.^4^^,^^7^^,^^8^^,^^12^

In fact, intra-articular injections of a γ-irradiated mixture of porcine type I atelopeptide collagen and polyvinylpyrrolidone showed effective results in 10 patients with knee OA.^7^^,^^8^ In addition, Martin Martin et al reported that a high molecular weight HA gave comparable clinical results in the treatment of 60 cases of knee OA using intra-articular injections of pure high molecular weight (300 kDa) type I collagen.^12^

However, the growing literature about hydrolyzed collagen highlights the major differences compared to native collagen. First, pure native collagen has a complex triple helical structure that needs specific enzymes to be incorporated into the ECM.^4^^,^^24^ In this context, the use of hydrolyzed collagen can be more efficient than native collagen in reinforcing the ECM and can contribute as a building block for the synthesis of collagen fibers by the cellular machinery.^4^^,^^24^ This is in agreement with the literature showing that hydrolyzed collagen induces a higher type II collagen biosynthesis in chondrocytes while native collagen is not able to support it.^4^^,^^15^ Second, hydrolyzed collagen, thanks to its low molecular weight and its simple composition, is more soluble and less viscous than native collagen.^1^^,^^9^^,^^10^ These features make the hydrolyzed collagen more bioavailable than native collagen.^1^^,^^9^^,^^10^

In comparison with native collagen, hydrolyzed collagen has another advantage, namely its antioxidative properties ^9^*,* which it extends toward the reactive oxygen species generated in rotator cuff tendinopathy ^25^^,^^26^.

The present study confirmed that CHondroGrid, as observed in the previous studies on knee OA, is efficient and safe in the treatment of rotator cuff tendinopathy and opens the possibility to the treatment of other types of tendinopathy in different anatomical districts.

Limitations of this study are the lack of a control group, the limited number of included patients, and the lack of post-treatment magnetic resonance imaging control.

Nonetheless, it is one of the largest studies on the periarticular injection of collagen and, more interestingly, it is the first one on the use of hydrolyzed collagen in RCT.

It would be interesting to evaluate the efficacy of hydrolyzed collagen in other tendinopathies as well as to compare its efficacy with other treatments.

## Conclusions

Results of the present study on 71 patients indicate that two periarticular injections of hydrolyzed collagen (CHondroGrid, Bioteck SpA, Arcugnano, Vicenza, Italy) administered about 10 days apart are safe and able to reduce pain and improve function at least in a short-term follow-up in the treatment of symptomatic rotator cuff tendinopathy.

Furthermore, controlled prospective studies including a higher number of patients should be carried out to compare hydrolyzed collagen effectiveness with other non-pharmacological treatments already available in the clinical practice.

## Disclaimers

Funding: No funding was disclosed by the authors.

Conflicts of interest: The authors, their immediate families, and any research foundation with which they are affiliated have not received any financial payments or other benefits from any commercial entity related to the subject of this article.
